# Feasibility and clinical experience of implementing a myoelectric upper limb orthosis in the rehabilitation of chronic stroke patients: A clinical case series report

**DOI:** 10.1371/journal.pone.0215311

**Published:** 2019-04-12

**Authors:** Jessica P. McCabe, Dennyse Henniger, Jessica Perkins, Margaret Skelly, Curtis Tatsuoka, Svetlana Pundik

**Affiliations:** 1 Brain Plasticity and NeuroRecovery Laboratory, Louis Stokes Cleveland Department of Veterans Affairs Medical Center, Cleveland, Ohio, United States of America; 2 Department of Physical Medicine and Rehabilitation, Louis Stokes Cleveland Department of Veterans Affairs Medical Center, Cleveland, Ohio, United States of America; 3 Department of Biostatistics, Case Western Reserve University, Cleveland, Ohio, United States of America; 4 Neurology Service, Louis Stokes Cleveland Department of Veterans Affairs Medical Center, Cleveland, Ohio, United States of America; 5 Department of Neurology, Case Western Reserve University School of Medicine, Cleveland, Ohio, United States of America; University of Tokyo, JAPAN

## Abstract

Individuals with stroke are often left with persistent upper limb dysfunction, even after treatment with traditional rehabilitation methods. The purpose of this retrospective study is to demonstrate feasibility of the implementation of an upper limb myoelectric orthosis for the treatment of persistent moderate upper limb impairment following stroke (>6 months). Methods: Nine patients (>6 months post stroke) participated in treatment at an outpatient Occupational Therapy department utilizing the MyoPro myoelectric orthotic device. Group therapy was provided at a frequency of 1–2 sessions per week (60–90 minutes per session). Patients were instructed to perform training with the device at home on non-therapy days and to continue with use of the device after completion of the group training period. Outcome measures included Fugl-Meyer Upper Limb Assessment (FM) and modified Ashworth Scale (MAS). Results: Patients demonstrated clinically important and statistically significant improvement of 9.0±4.8 points (p = 0.0005) on a measure of motor control impairment (FM) during participation in group training. It was feasible to administer the training in a group setting with the MyoPro, using a 1:4 ratio (therapist to patients). Muscle tone improved for muscles with MAS >1.5 at baseline. Discussion: Myoelectric orthosis use is feasible in a group clinic setting and in home-use structure for chronic stroke survivors. Clinically important motor control gains were observed on FM in 7 of 9 patients who participated in training.

## Introduction

Stroke is a leading cause of long term disability in the United States[1]. Traditional rehabilitation does not restore normal motor control for all stroke survivors, and upwards of 50% live with persistent upper limb dysfunction[2]. This leads to diminished functional independence and quality of life[3]. Motor learning-based interventions have shown promise[4]. However in today’s health care milieu, for those with chronic motor deficits, provision of the intensive rehabilitation necessary to provide motor learning-based interventions is challenging. Therefore, new treatment methods are needed under these constraints.

An emerging technology that warrants further investigation is myoelectric control which harnesses the user’s EMG signal to power a custom fabricated orthotic device. When the user activates a target muscle, the EMG signal from that muscle signals a motor to produce a desired movement. Myoelectric control has been studied in different populations[5], but its study in stroke has been limited. One commercially available upper limb myoelectric device is the MyoPro motion-G (Cambridge, MA). The MyoPro motion-G provides assistance to the weak upper limb and allows the patient to perform movement they otherwise are unable to complete. Preliminary evidence suggests it may be effective in improving motor control[6–9] and one study showed improvement in self-reported function and perception of recovery[10]. This device has been utilized in the occupational therapy (OT) clinic at our medical center for 5 years. The purpose of this study is to demonstrate feasibility of administering treatment with the MyoPro using a group therapy design in a cohort of patients with chronic stroke whose progress with standard OT had plateaued.

## Methods

This is a retrospective analysis of data collected longitudinally while chronic stroke patients participated in group training with a MyoPro in our clinic. Training was provided by OT staff. This study was approved by the IRB of the Louis Stokes Cleveland Department of Veterans Affairs Medical Center (IRB #17030-H23). Approval was obtained to review and analyze patient data.

### MyoPro treatment candidate selection criteria

Patients were assessed for eligibility to receive a MyoPro once they reached a plateau in functional performance following traditional OT. Inclusion criteria included: regular/consistent therapy attendance; trace muscle contraction in major upper limb muscle groups; adequate passive ROM to don/doff device; intact cognition; active shoulder flexion ≥40° and shoulder abduction ≥20°; ability to don/doff device with/without a reliable caregiver. Nine patients were prescribed a MyoPro at the conclusion of their regular OT.

### Technology

**Fig 1:**
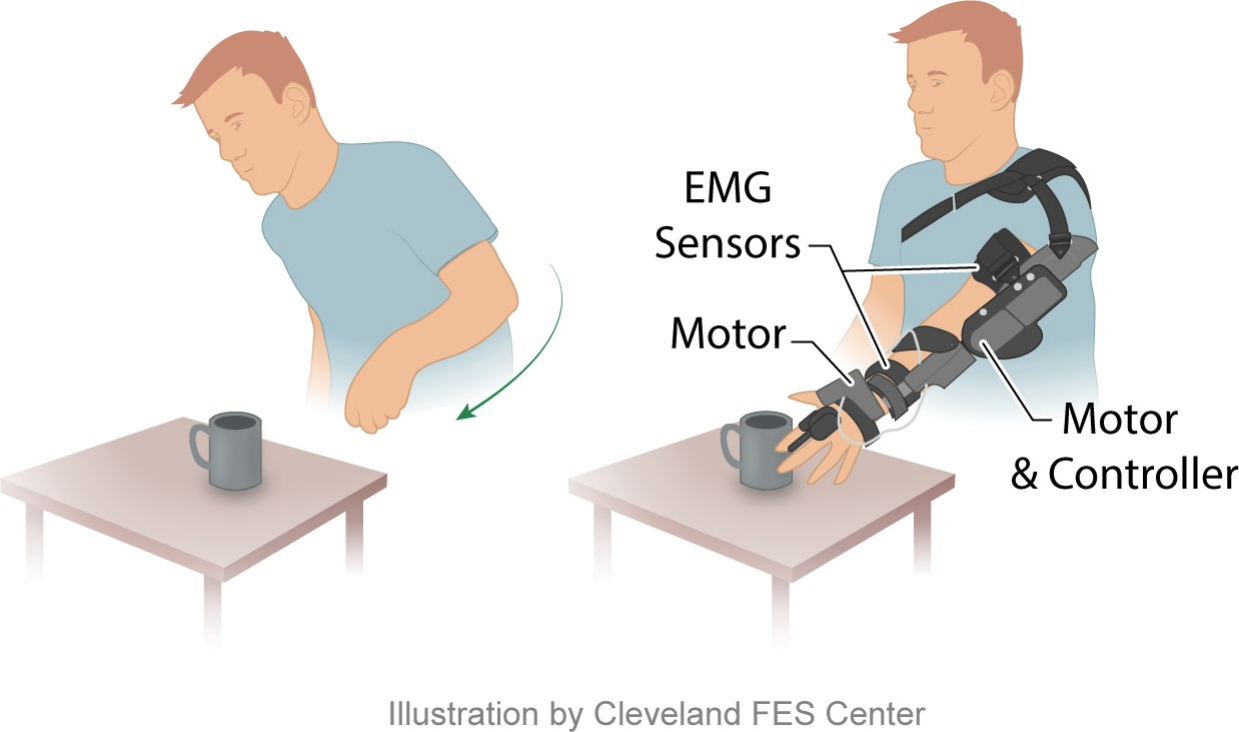
Schematic drawing of an individual reaching for an object without and with the device donned. With assist of the device, the goal directed movement of reaching for the object can be completed. The Cleveland FES Center created this illustration and has granted the authors permission to publish it in this manuscript.

The MyoPro Motion-G is a custom fabricated, myoelectric upper limb orthosis worn on the paretic upper limb (Fig 1). It supports the affected limb and assists the user to perform flexion/extension of the elbow and opening/closing of the hand. Sensors within the device detect the patient’s EMG signal during volitional muscle contraction. When an EMG signal is sensed, motors within the device provide assistance to complete the desired movement (i.e. hand opening/closing). Using computer software, the therapist adjusts the EMG level at which device movement is triggered. The degree of movement produced by the device is proportional to the recorded EMG level produced by the patient’s volitional effort. Patients interface with the device through a push button control panel and via software on a computer. Users can switch between 4 individual SINGLE modes (BICEP mode, TRICEP mode, hand CLOSE mode, and hand OPEN mode), and are able to train multi-joint movement by practicing combinations of these modes (i.e. BICEP+hand CLOSE modes). Additionally, users can combine both EMG sensors simultaneously at a given joint (i.e. DUAL mode elbow, which combines the biceps and triceps EMG sensors; DUAL mode hand, which combines the finger flexor and extensor EMG sensors) or all 4 EMG sensors can be used simultaneously (DUAL mode elbow + DUAL mode hand). To practice joint movement in a SINGLE mode, the patient is required to produce an adequate EMG signal to reach the therapist-adjusted threshold. To return to the starting position their EMG must drop below this threshold (i.e. the patient must learn to relax the muscle). To trigger movement in DUAL mode, both flexor and extensor EMG signals are taken into account. The motor is activated after a corresponding muscle’s EMG exceeds its threshold and is greater than the EMG in the antagonist muscle. For example, elbow extension is achieved when triceps EMG exceeds its threshold and biceps EMG is less than triceps EMG. This practice facilitates relearning coordinated control of agonist/antagonist muscles across the joint as opposed to abnormal co-activation of both muscles which precludes practice of such movement.

### Intervention

An orthotist performed custom fabrication of each patient’s device along with initial fitting and setting of device parameters for training. A group training paradigm was employed where patients were scheduled for 1–2 weekly group sessions (60–90 minutes/session). Patients unable to attend the group sessions received individualized MyoPro training (n = 2).

#### Patient selection

Patients entered the group when they were deemed plateaued in their traditional therapies. The group accepted new patients using a rolling enrollment schedule-i.e. an individual was offered entry into the group when they were plateaued with their traditional OT. Because of this, patients joined the group at different time points and therefore some have data that spans a longer duration. Upon discontinuation of group therapy, patients were issued a home exercise program to complete with their MyoPro and encouraged to use the device in performing everyday activity. In this data analysis, we labeled the first phase (i.e. when they attended group therapy training) as the Supervised phase and the following phase as the Unsupervised phase (when group therapy was discontinued). For the Unsupervised phase, all patients had access to their device as it was purchased for their personal use. However, 5/9 patients did not attend any further therapy sessions after the group training ended and therefore received no further evaluation. Four patients had sought out additional visits with OT (1 session every 5–10 weeks) after the conclusion of the group therapy and were subsequently re-evaluated. It is notable that some patients did not have a device for some period of time during this Unsupervised period as it was being upgraded to the most current model.

#### Intervention: Training progression

At each session, patients donned the device. Then, a training progression was employed (Table 1). First, patients performed preparatory exercises using SINGLE modes (i.e. BICEP mode or hand CLOSE mode). During BICEP mode training, patients were instructed to bend their elbow and then relax back to the start position. For hand CLOSE mode, they practiced closing their hand and relaxing back to the start position. After preparatory activities, therapeutic exercises were performed in either the seated or standing position. These exercises were designed to prepare patients for activities that required sustained contraction or contraction across multiple joints in preparation for function. For example, patients performed multiple repetitions of BICEP mode with instruction to bend their elbow, sustain a position for a fixed time, and then relax the elbow followed by completing hand CLOSE mode, holding for a period of time and relaxing. Depending on individual ability, exercises also included combined hand and elbow motions to work on separate and simultaneous elbow and hand motions. As patients progressed, they would also perform functional task training with the device including sorting laundry, placing utensils away, sorting tools, holding a pot while stirring with unaffected hand, and sweeping.

**Table 1:** Summary of group training protocol.

1. Don on Device 2. Warm Ups/Preparatory Activities (Sitting &/or Standing) a. Bend elbow, relax/extend– 25 reps (BICEP or DUAL MODE) b. Extend elbow, relax/flex– 25 reps (TRICEP or DUAL MODE) c. Close hand, relax/open– 25 reps (CLOSE or DUAL MODE) d. Open hand, relax/close– 25 reps (OPEN or DUAL MODE) 3. Therapeutic Exercises (Seated &/or Standing) a. Bend elbow and Hold 10+ seconds, then Relax– 25 reps b. Close Hand and Hold 10+ seconds, then Relax– 25 reps c. Yo-yos: Bend elbow low, middle, full (~45*, 90*, full range)– 25 reps **relax and extend elbow between each rep d. Combined motions: i. Close hand>Bend Elbow>Open Hand>Extend Elbow– 25 reps ii. Open hand>Bend Elbow>Close Hand> Extend Elbow>Open Hand– 25 reps 4. Functional Tasks a. Sorting tools, laundry, utensils 5. Turn off/doff Device

#### Outcomes

Outcome measures were the Fugl Meyer Upper Limb Assessment (FM) and modified Ashworth Scale (MAS). FM is an impairment measure of motor control, [11] with good validity,[12] intra-rater and inter-rater reliability,[13] and is recommended for use in chronic stroke trials[14] (0–66 points; a higher score equals less impairment). MAS is a commonly used clinical test of muscle tone with high interrater reliability (kappa = 0.92 or percent of agreement = 97.4%)[15]. It consists of a six-point scale (0, 1, 1.5, 2, 3 and 4) used to grade tone elicited during passive movement[16]. A score of 0 corresponds to normal tone while a rating of 4 corresponds to rigidity. The timing of data collection varied among patients. However, they were all evaluated at around the 12-week time point of the device use. Outcomes were collected without the device donned.

#### Statistical analysis

Data analysis included descriptive statistics and use of paired t-tests. Two-sided Type I error level of 0.05 was adopted for hypothesis testing.

## Results

**Fig 2:**
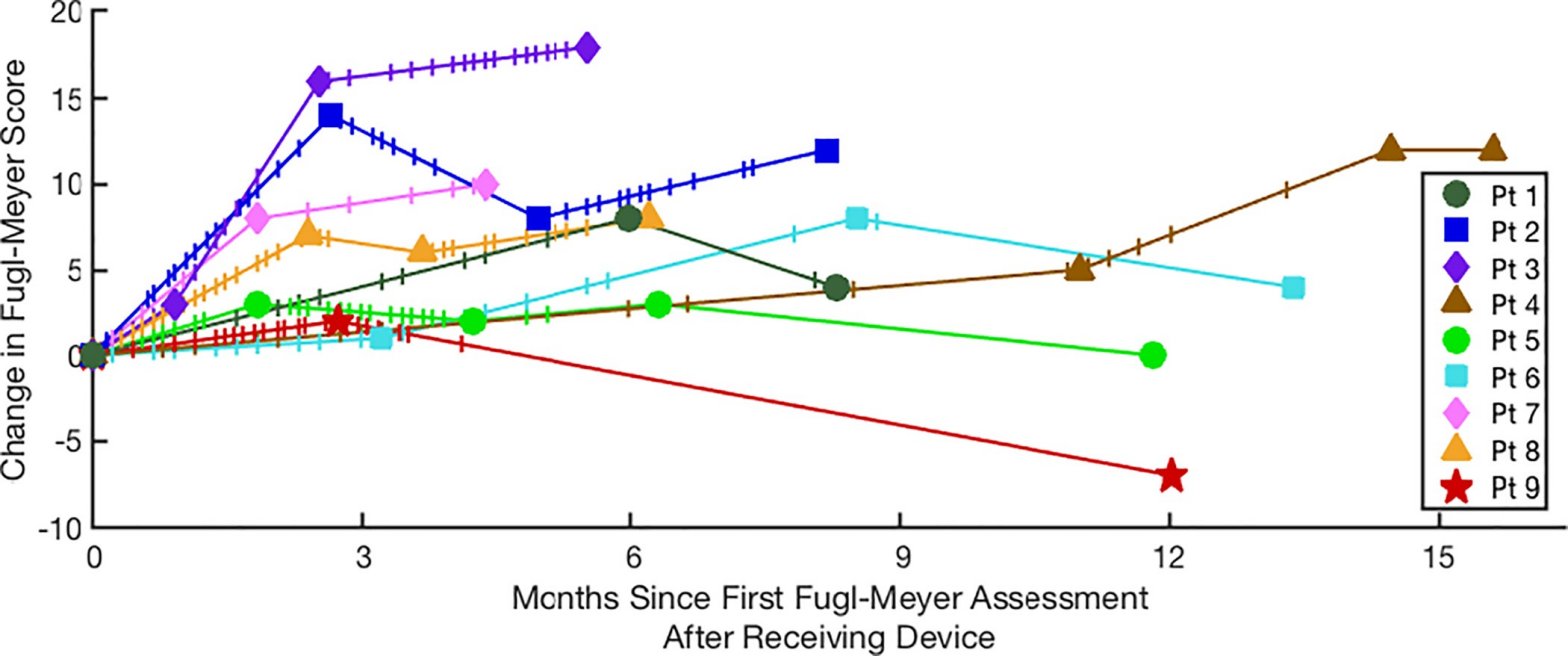
Change in Fugl-Meyer score over time for each group therapy participant. The change in FM score from the initial evaluation is shown with different symbols for each patient. Vertical tick marks correspond to therapy sessions using the device for each individual of the group.

Table 2 provides patient characteristics (n = 9). Fig 2 provides information regarding the change in FM score over time along with individual patient participation patterns in the group training sessions. Patients were moderately impaired at initial data collection according to FM (Table 3). Four of 9 patients had their dominant arm affected by stroke (patients 1,2,3 and 9, Table 3).

**Table 2:** Patients’ Characteristics (N = 9).

Age in years, mean (SD)	62 (9.5)
Female, %	11%
Years since stroke, mean (SD)	4.3 (3.7)
Stroke Hemisphere, % Left	67%
Stroke Type, % ischemic	78%

**Table 3:** Device use and change in Fugl-Meyer scores.

		Supervised Phase					Unsupervised phase	
Patient	Initial FM	12-week testing (actual week #)	FM change @ 12-week testing	Supervised phase duration (weeks)	FM change during Supervised phase	Unsupervised phase duration (weeks)	FM change post Supervised phase	FM change from initial test
1	24			26.0	+8	10.0	-4	+4
2	24	11.6	+14	35.6	+12	na		+12
3	29	11.0	+16	24.0	+18	na		+18
4	41			62.9	+12	na		+12
5	53	8.0	+3	27.4	+3	24.0	-3	0
6	29	14.0	+1	37.0	+8	21.1	-4	+4
7	42	8.0	+8	19.0	+10	na		+10
8	14	10.4	+7	27.0	+8	na		+8
9	45	11.9	+2	11.9	+2	40.4	-9	-7
Mean (SD)	33.4 (12.5)	10.7 (2.1)	7.3 (5.9)*	30.1 (14.5)	+9.0 (4.8)^†^	24.0 (12.6)	-5.0 (2.7)	+6.8 (7.4)^‡^

* t-test p = 0.017

^†^ t-test p = 0.00053

^‡^ t-test p = 0.026

na–not applicable as these patients did not have re-evaluation following an Unsupervised phase

### Supervised phase: change in FM score

There was variability in the length of time patients trained with the device during the Supervised phase (11.9–62 weeks), however, the majority of patients (7/9) had testing completed around the 12-week point of using the device (Table 3 and Fig 2). Two patients were not re-tested until the 26^th^ (patient#1) and 62^nd^ (patient#4) weeks of participation in the MyoPro group therapy. For those who were evaluated at about 12 weeks of working in the Supervised phase, there were significant changes in FM (7.3 (5.9) points; p = 0.017; Table 3). The timing of 12-week testing varied from 8 to 14 weeks. At the conclusion of the Supervised phase, patients had participated in group training an average of 32.16(12.8) hours and a statistically significant change from the initial FM score was observed (9.0(4.8) points; p = 0.00053; Table 3). Sixty percent of patients improved FM between the 12-week testing and the end of the Supervised phase (mean length of Supervised phase was 30 weeks). Seven out of nine patients demonstrated a FM change score ≥5 points at the end of the Supervised phase which is within or above the minimal clinically important difference (MCID) range of 4.25–7.25 points[17]. Patient#7 had non-device OT sessions twice per week in addition to the group device visits.

### Unsupervised phase: FM score

Only 4 patients had FM scores obtained following the Unsupervised phase (Table 3 and Fig 2). These 4 patients demonstrated some worsening, although most were still improved compared with the initial score. Patient#1 had initial FM = 24, gained 8 points during the Supervised phase, but lost 4 FM points during the Unsupervised period even though they were still attending near-weekly standard OT sessions without the device. Patient#5 had initial FM = 53, gained 3 and then lost 3 FM points. However, patient#5 had additional twice weekly OT sessions without the device for the 1^st^ half the Supervised phase and weekly sessions for the 2^nd^ half. During the Unsupervised phase this patient did not use the device half the time due to device upgrading and participation in a research study. Patient#6 had initial FM = 29, gained 8 and then lost 4 FM points during the Unsupervised phase; this patient also had biceps, forearm and hand botulinum toxin treatments for spasticity during the Unsupervised phase. Patient#9 had initial FM = 45, gained 2, but lost 9; the patient did not have the device for 2 months prior to the final evaluation.

### Change in MAS score

At the initial testing, abnormal muscle tone (MAS>0) was detected in elbow flexors of 6 patients and in wrist flexors of all tested patients (Table 4). Data is missing for patient#7 (initial test) and patient#4 (after device use). Although there was no statistically significant pre to post change observed with a group-wise comparison (p>0.05), improvements were observed for some patients. For elbow flexor tone, there was an improvement of MAS score for 3 patients and worsening in 1 patient. The improvement for patient#6 from 1.0 to 0 was made approximately 4 months after receiving the device and 9 months prior to reporting initiation of botulinum toxin treatments. Worsening of MAS score occurred in patient#5 who had very mild MAS of 1 at the initial test. For wrist flexors, there was an improvement in MAS score for 4 patients and worsening in one. Importantly, for 3 patients with MAS > 1.5 at initial testing, there was a consistent improvement in MAS score during the Supervised phase.

**Table 4:** Modified Ashworth scale scores before and after device use.

	Elbow Flexors			Wrist Flexors			
Patient	Pre*	Post	Change	Pre	Post	Change	Time with MyoPro (weeks)^†^
1	0.0	0.0	0.0	1.0	1.0	0.0	46.0
2	4.0	1.5	-2.5	4.0	1.5	-2.5	47.0
3	0.0	0.0	0.0	1.5	1.0	-0.5	27.1
4	1.0	NC		1.0	NC		
5	1.0	1.5	0.5	2.0	1.5	-0.5	4.3
6	1.0	0.0	-1.0	1.0	1.0	0.0	17.0
7	NC	1.0		NC	0.0		40.1
8	1.5	1.5	0.0	1.0	1.5	0.5	39.0
9	2.0	1.0	-1.0	3.0	2.0	-1.0	19.4
Mean (SD)	1.3 (1.3)	0.8 (0.7)	-0.6 (1.0)	1.8 (1.1)	1.2 (0.6)	-0.6 (1.0)	30.0 (15.5)

NC = not collected

* MAS prior to receiving the MyoPro

^†^ Time elapsed after receiving the MyoPro

## Discussion

This study provides evidence that it is feasible to utilize a myoelectric upper limb orthosis using a group training paradigm for the rehabilitation of moderately impaired chronic stroke survivors. The main finding is that clinically important changes on a motor control performance measure were observed in individuals with chronic stroke who participated in group training. Of note, these patients were deemed plateaued with traditional OT services and were being discharged from standard care. There was a trend toward decreased flexor tone in individuals presenting with elevated flexor tone.

The gains on FM in our clinical practice setting study are comparable to many studies conducted in the research setting with subjects who were less impaired. For example, in research studies of less impaired individuals (baseline FM 39–55 points), patients demonstrated gains ranging from 6–9 points on FM[18–26]. For studies with similar impairment level to our cohort (baseline FM 27–36 points), treatment gains on FM ranged from 2–14 points[27–35]. Participants across these research studies trained an average of 24.9 hours (range of 7–48 hours), similar to the number of face to face hours for our patient cohort. It is encouraging that our results are comparable to many of these studies even though they were obtained in a clinical treatment setting under the constraints of current health care delivery where patient selection and therapy administration is less strictly controlled than in the rehabilitation administered within the research setting. Translation of research into actual clinical practice is an area of great interest and significant challenge in rehabilitation science[36,37]. Within the research setting, rehabilitation typically has strict subject inclusion/exclusion criteria, duration, intensity and content of care. Our patient cohort reflects real world variability in impairment levels and the care that is specifically tailored to meet the needs of the individual receiving the care (as opposed to a standardized research intervention). Our observation, therefore, presents a novel training paradigm that would be feasible within our current clinical practice setting using a group therapy approach and motor learning as the basis for intervention.

The MyoPro allows practice of three important motor learning principles. First, it encourages coordinated, volitional muscle activation. With EMG-biofeedback the patient learns to selectively contract a desired muscle in a coordinated manner that would be difficult to accomplish without the device. Often when a patient attempts a movement (i.e. grasp preparation), involuntary co-contraction of the antagonist occurs. This abnormal co-contraction limits opening of the hand in preparation for grasp. Furthermore, due to disparity of muscle strength between the agonist/antagonist pairings (i.e. greater strength in the finger flexors versus the extensors), the ability to selectively practice finger extension is precluded. Others have studied whether myoelectric control can address pathological co-contraction with some promising findings[38,39]. In one study, EMG biofeedback training was used to decouple the anterior deltoid and bicep muscles, and patients demonstrated more selective muscle activation with modest improvement on FM[38]. Improved coordination of agonist/antagonist pairs was also demonstrated with a hybrid EMG-driven robotic/neuromuscular electrical stimulation system and there was a clinically significant improvement on FM[39]. The Myopro employs biofeedback in a targeted manner to allow for very consistent, incremental practice of individual muscle activation as well as coordination training of agonist/antagonist pairings. The second principle is motivation for repeated practice which is encouraged by the reward of movement the device delivers. Moderately/severely impaired patients are usually discouraged in their repeated task practice because they do not experience the product of their hard labor. This device compensates for patients’ physical disabilities and produces a desired joint movement which motivates additional practice. Furthermore, the training is ultimately aimed at completion of meaningful functional tasks which provides further motivation. Finally, the device allows for incremental progression of training. Both single and multi-joint movement exercises can be performed; exercise can be fragmented; device settings (i.e. EMG threshold) can be adjusted to introduce additional challenge; and practice can be done in different body postures. Overall, the combination of EMG biofeedback with a wearable powered upper limb orthosis presents a powerful therapeutic tool that fits well within the framework of motor learning.

Patients were scheduled for 1–2 weekly sessions. However, high attendance variability was noted with some patients (i.e. patient#4; Fig 2) while others attended a greater number of sessions within a shorter time frame (i.e. patient# 3; Fig 2). Despite this variability, patients improved individually on the main study outcome measure. It is reasonable to suggest that consistent gains across all participants may have occurred because patients had access and were encouraged to practice with their device in the home setting.

Our study results were obtained in a clinical setting using a group therapy training paradigm. This is important for a few reasons. First, our study demonstrates that clinically important treatment gains can be made in chronic stroke patients with persistent motor control deficits in an actual clinical setting, as opposed to a controlled research laboratory. Furthermore, training was implemented efficiently in a group training paradigm (4:1 patient to therapist ratio). Delivery of care in a group setting allows more patients to benefit from limited rehabilitation resources and evidence suggests it can be as effective as individual therapy in stroke[40]. Additionally with group therapy training, patients were given the opportunity to train over several months. This may have allowed sufficient time to address persistent motor control impairments and allow for consolidation of gains. Of note, patients in our cohort had plateaued in traditional OT, and with exception of a few were being discharged from therapy. However, they were given the opportunity to try this novel training approach. Clinically important and statistically significant gains were made on the FM after therapists assessed the patients to be plateaued with traditional services. Though efficacy of group training with the device cannot be determined due to small sample size and variability of training parameters, our results provide groundwork for further examination of this training paradigm to be considered in addressing persistent deficits following stroke.

Mitigation of elevated flexor muscle tone was demonstrated in several patients. Three patients showed a decrease in elbow flexor tone and 4 patients showed a decrease in wrist flexor tone by the end of the study. The variability in the muscle tone data is likely due to small sample size and/or inclusion of patients who did not initially present with tone or presented with mildly increased tone. Furthermore, muscle tone management may require regular use of the device. Future research will be needed to evaluate the effect of myoelectric orthotics on management of spasticity.

Data for the Unsupervised phase is limited in two ways. First, only 4 of 9 patients were re-evaluated. Second, there were long periods when patients did not have full access to their device as it was being upgraded. As a result, much of the time participants were without their device and could not continue their independent training. During the Unsupervised phase, we observed degradation in motor control performance according to FM for all patients. Non-use of the device may result in deterioration of motor performance, although this conclusion is preliminary. Our results suggest that further study is warranted to determine whether regular home use of the device is needed to maintain gains made during supervised training.

Several limitations of the current study constrain interpretation of our findings. Two main limitations were the inconsistent timing with which testing was completed and variability in treatment doses across different patients. Additionally, this was a retrospective analysis of clinical care delivered to a small, heterogeneous group of stroke survivors and data was not available on patients’ adherence with the home exercise program. However, given that this was an actual clinical setting and not a designed clinical trial, our data may be more representative of clinical practice patterns in chronic stroke. Finally, we report only measures of impairment, thus limiting our interpretation of findings in terms of function and quality of life. More robust measurement across multiple domains is necessary to further elucidate how the device impacts patient care and functional performance.

## Conclusions

In a group clinical setting, it was feasible to implement a myoelectric upper limb orthosis with chronic stroke survivors. Clinically important and statistically significant gains were made on a measure of upper limb motor control. The results may be explained by the motor learning based functionality of the device. Further study is warranted in a larger cohort.

## Abbreviations

FM: Fugl Meyer Upper Limb Assessment
MAS: Modified Ashworth Scale
EMG: Electromyography
OT: occupational therapy
MCID: minimal clinically important difference
